# Chloroplast genome characteristics and phylogenetic analysis of the medicinal plant *Blumea balsamifera* (L.) DC

**DOI:** 10.1590/1678-4685-GMB-2021-0095

**Published:** 2021-11-15

**Authors:** Chao Zhao, Wenfen Xu, Yuan Huang, Qingwen Sun, Bo Wang, Chunlin Chen, Qiyu Chen

**Affiliations:** 1 Guizhou University of Traditional Chinese Medicine, College of Pharmacy, Guiyang, China.

**Keywords:** Blumea balsamifera, chloroplast genome, codon usage, repeat sequence, phylogenetic analysis

## Abstract

*Blumea balsamifera* (L.) DC., a medicinal plant with high economic value in the *Asteraceae* family, is widely distributed in China and Southeast Asia. However, studies on the population structure or phylogenetic relationships with other related species are rare owing to the lack of genome information. In this study, through high-throughput sequencing, we found that the chloroplast genome of *B. balsamifera* was 151,170 bp in length, with a pair of inverted repeat regions (IRa and IRb) comprising 24,982 bp, a large single-copy (LSC) region comprising 82,740 bp, and a small single-copy (SSC) region comprising 18,466 bp. A total of 130 genes were identified in the chloroplast genome of *B. balsamifera*, including 85 protein-coding, 37 transfer RNA, and 8 ribosomal RNA genes; furthermore, sequence analysis identified 53 simple sequence repeats. Whole chloroplast genome comparison indicated that the inverted regions (IR) were more conserved than large single-copy and SSC regions. Phylogenetic analysis showed that *B. balsamifera* is closely related to *Pluchea indica*. Conclusively, the chloroplast genome of *B. balsamifera* was helpful for species identification and analysis of the genetic diversity and evolution in the genus *Blumea* and family *Asteraceae*.

## Introduction

*Blumea balsamifera* (L.) DC. is a perennial herb or subshrub that belongs to the family *Asteraceae* and is mainly distributed in China, India, Thailand, and the Philippines (Chinese Academy of Sciences editorial commission of the flora, 1988). As a traditional ethnic Miao’s medicinal plant in China, *B. balsamifera* is commonly called Ai-Na-Xiang and Da-Feng-Ai and is extensively used to treat eczema, dermatitis, rheumatism, and wind syndrome of the head (Dewen, 2005). In the Philippines, this plant is called Sambong, a leading prescribed medicine for diuresis and antiurolithiasis (Toralba et al., 2015). Recent studies have demonstrated that this plant possesses several pharmacological activities, such as antitumor (Hasegawa et al., 2006) and anti-inflammatory (Sakee et al., 2011). Additionally, because of its strong aromatic properties, the leaves of the plant are used as flavoring ingredients and tea (Xu et al., 2012), which have great economic value and attract the attention of researchers.

*Asteraceae* is the largest family of flowering plants with numerous species, including 1,479 genera and 21,105 species. It is widely distributed in the world except the Antarctic region (Dempewolf et al., 2008). *Blumea* is a highly complex genus in the *Asteraceae* family. It was first placed in the tribe Astereae and later moved to the tribe Inuleae owing to the characteristics of anthers. In 1989, Anderberg separated the tribe Gnaphalieae from the tribe Inuleae based on the morphological characteristics of plants and confirmed the accuracy of the classification based on the results of *ndhF* sequencing analysis (Anderberg *et al.*, 1991, 2005). However, the above classification based on plant morphology and short DNA sequences has limitations; therefore, more information needs to be collected for analysis and discussion. Chloroplasts are important organelles in green plants which mainly participate in protein, pigment, and starch biosynthesis (Rodriguez-Ezpeleta et al., 2005). Additionally, the chloroplast genome has fewer nucleotide substitutions and rearrangement of genome structure than the nuclear genome (Smith, 2015). The genome size, content, and structure are more conserved in the chloroplast genome than in the nuclear genome (Wicke et al., 2011). Therefore, it becomes an ideal model for studying genome evolution, phylogenetic analysis, and species identification in complex angiosperm families. However, the chloroplast genome of *Blumea* has not yet been reported, which is inconducive to further study on the evolutionary history and status of *Blumea*.

Therefore, this study adopted Illumina sequencing technology to sequence the whole chloroplast (cp) genome of *B. balsamifera* and compared it with other available *Asteraceae* species to explore their genetic divergence, genetic structural characteristics, and phylogenetic relationships. Thus, our study provides valuable information for elucidating the evolution of *B. balsamifera*, developing molecular markers, and revealing phylogenetic relationships in the *Asteraceae* family.

## Material and Methods

### Plant material, DNA extraction and sequencing

Fresh leaves of *B. balsamifera* were collected from Hongshuihe Town, Luodian County, Guizhou Province, China (25°09′56.8″N, 106°37′24.1″E) and stored in liquid nitrogen. The voucher specimens were deposited in the Center of Herbarium, Guizhou University of Traditional Chinese Medicine, China, under accession number WB20191003. Total DNA was extracted from leaf tissue of *B. balsamifera* using the EZNA Plant DNA extraction kit (OMEGA, USA). Subsequently, DNA purity and quantity were evaluated using NanoPhotometer spectrophotometer (IMPLEN, USA) and Qubit 2.0 Fluorometer (Life Technologies, USA), respectively. A genomic library was constructed using the TruSeq Nano DNA Sample Prep Kit (Illumina, USA), following the manufacturer’s protocol. Samples were sequenced on Illumina NovaSeq (Illumina, USA) platform, and 150 bp paired-end reads were generated. The raw reads were deposited in the NCBI sequence read archive with the accession number PRJNA728381.

### Chloroplast genome assembly and annotation

First, the raw reads were filtered using the Trimmomatic (Bolger et al., 2014) software. Next, the cp genome was assembled using the NOVOPlasty (Dierckxsens et al., 2017) software. The Gap Closer (Luo et al., 2012) software was used to repair the inner gaps of the assembly results. Additionally, considering the genome sequence of *Pluchea indica* (NC_038194.1) as a reference, the whole cp genome of *B. balsamifera* was annotated by DOGMA (Wyman et al., 2004). The structure of transfer RNA (tRNA) genes was determined using the tRNAscan-SE (Lowe and Chan, 2016) online software, and the whole cp genome map was constructed using OGDRAW v.1.2 (Lohse et al., 2007). The cp genome sequence of *B. balsamifera* was uploaded to the GenBank database with the accession number MW769705.

### Codon usage analysis and repeat sequence detection

MEGA v.7.0 (Kumar et al., 2016) was used to analyze the synonymous codon usage and the relative synonymous codon usage (RSCU) of the *B. balsamifera* cp genome. MISA (Beier et al., 2017) software was used to detect simple repetitive sequences (SSRs) in the cp genome of *B. balsamifera* with the minimum repetitive unit set as follows: single nucleotide > 10, dinucleotide > 5, trinucleotide > 4, tetranucleotide > 3, pentanucleotide > 3, and hexanucleotide > 3. The long repeats in *B. balsamifera* were identified using the REPuter software (Kurtz et al., 2001). The minimum repeat length was set to 30 bp, and the similarity between repeat sequences was >90%. 

### Comparative analysis of chloroplast genomes

The whole cp genome differences in *B. balsamifera* were compared with related species; cp genomes of four *Asteraceae* plants (*Anaphalis sinica*, NC_034648.1; *Leontopodium leiolepis*, NC_027835.1; and *Helichrysum italicum*, NC_041458.1; *P. indica*, NC_038194.1) were obtained from GenBank. Additionally, the mVista (Frazer et al., 2004) program with Shuffle-LAGAN mode compared the genome of *B. balsamifera* cp with those of other selected species. 

### Phylogenetic analysis

Cp genomes of 37 *Asteraceae* plants and three *Rosaceae* plants were downloaded from the NCBI database, and three Rosaceae plants were set as the outgroup (Table S1). The phylogenetic reconstruction analysis was performed using the RAxML program (Stamatakis, 2014) with the maximum likelihood (ML) method. Sequence comparison was completed using the MAFFT software (Katoh and Standley, 2013), and necessary manual inspection and result adjustment on Se-Al v.2.04 (http://tree.bio.ed.ac.uk/software/). Moreover, GTR + I + G was selected as the nucleotide substitution model. Finally, the bootstrap values (BS) of each branch of the phylogenetic tree were obtained by 1000 self-expanding repeat analysis.

## Results and Discussion

### Chloroplast genome features of B. balsamifera

**Figure 1 -:**
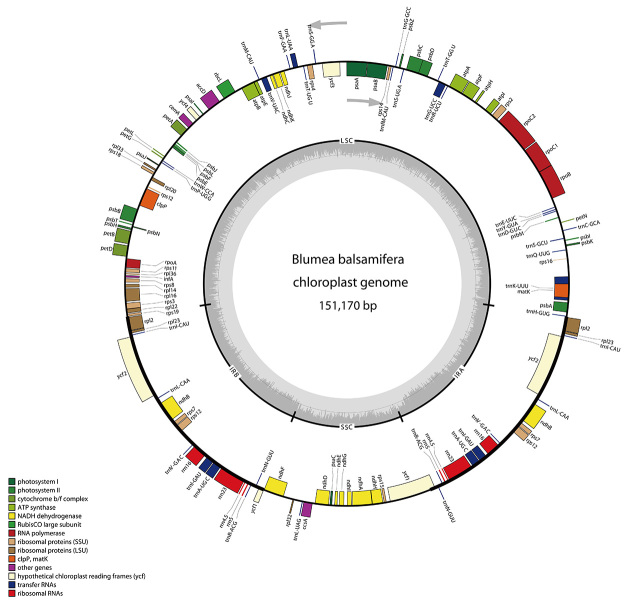
Gene map of the complete cp genome of *Blumea balsamifera*. Genes present within the circle are transcribed clockwise and those outside are transcribed counterclockwise. Genes are color-filled based on different functions. Inverted repeat (IR), small single-copy (SSC), and large single-copy (LSC) regions are indicated.

The complete cp genome of *B. balsamifera* is 151,170 bp in size, which has a typical quadripartite structure and harbors a pair of inverted repeat regions (IRa and IRb) of 24,982 bp in size, separating the large single-copy (LSC) region of 82,740 bp from the small single-copy (SSC) region of 18,466 bp (Figure 1). The GC content of the complete cp genome, LSC, SSC, and IR regions are 37.50%, 35.80%, 31.10%, and 43.00%, respectively (Table S2). We found that the GC content in the IR region was the highest, whereas that in the SSC region was the lowest. Related studies have demonstrated that frequent transformation of GC-biased genes in the IR region is the main reason for the high GC content in the IR region (Wu and Chaw, 2015). Overall, 130 genes were annotated, including 85 protein-coding, 37 tRNA, and 8 ribosomal RNA (rRNA) genes (Table S2). Among these genes, 4 rRNA, 7 tRNA, and 6 protein-coding genes were duplicated in the IR region (Table 1). Furthermore, we found that nine protein-coding genes and seven transporter RNA genes contain a single intron and three protein-coding genes have two introns (Table 1). Additionally, *rps12* is a trans-splicing gene with the 5′-exon located in the LSC region and 3′-end exon duplicated in the IRs, which is common among plant cp genomes.

**Table 1 -:** Genes present in the chloroplast genomes of *Blumea balsamifera*.

Category	Group of Genes	Name of Genes
Self-replication	Small subunit of ribosome (SSU)	rps2, rps3, rps4, rps7(2), rps8, rps11, rps12^**^(2), rps14, rps15, rps16^*^, rps18, rps19
	Large subunit of ribosome (LSU)	rpl2^*^(2), rpl14, rpl16^*^, rpl20, rpl22, rpl23(2), rpl32, rpl33, rpl36
	DNA dependent RNA polymerase	rpoA, rpoB, rpoC1^*^, rpoC2
	Ribosomal RNA(rRNA)	*rrn4.5(2), rrn5(2), rrn16(2), rrn23(2)*
	Transfer RNAs (tRNA)	trnA-UGC^*^(2), trnC-GCA, trnD-GUC, trnE-UUC, trnF-GAA, trnfM-CAU, trnG-GCC^*^, trnG-UCC^*^, trnH-GUG, trnI-CAU(2), trnI-GAU^*^(2), trnK-UUU^*^, trnL-CAA(2), trnL-UAA^*^, trnL-UAG, trnM-CAU, trnN-GUU(2), trnP-UGG, trnQ-UUG, trnR-ACG(2), trnR-UCU, trnS-GCU, trnS-GGA, trnS-UGA, trnT-GGU, trnT-UGU, trnV-GAC^*^(2), trnV-UAC, trnW-CCA, trnY-GUA
Photosynthesis	Photosystem I	*psaA, psaB, psaC, psaI, psaJ*
	Photosystem II	*psbA, psbB, psbC, psbD, psbE, psbF, psbH, psbI, psbJ, psbK, psbL, psbM, psbN, psbT, psbZ*
	Subunits of NADH-dehydrogenase	ndhA^*^, ndhB^*^(2), ndhC, ndhD, ndhE, ndhF, ndhG, ndhH, ndhI, ndhJ, ndhK
	Subunits of cytochrome b/f complex	petA, petB^*^, petD^*^, petG, petL, petN
	Subunits of ATP synthase	atpA, atpB, atpE, atpF^*^, atpH, atpI
	Large subunit of rubisco	*rbcL*
Other genes	ATP-dependent protease subunit p gene	clpP^**^
	Translational initiation factor	*infA*
	Maturase	*matK*
	Envelope membrane protein	*cemA*
	Subunit of acetyl-CoA-carboxylase	*accD*
	C-type cytochrome synthesis gene	*ccsA*
Unknown function	Hypothetical chloroplast reading frames (ycf)	ycf1, ycf2(2), ycf3^**^, ycf4

(2) indicates the number of the repeat unit is 2; ^*^genes with one intron; ^**^genes with two introns.

### Codon usage analysis

Codon bias plays a key role in translation and controls protein production and folding (Quax et al., 2015). This study found that 85 protein-coding genes in *B. balsamifera* genome are encoded by 64 codons; three are stop codons (UAA, UGA, UAG, Table S3). Among the 20 amino acids, leucine (10.65%) accounts for the largest proportion and cysteine (1.11%) accounts for the smallest (Figure S1). In other angiosperm cp genomes, the reported leucine and cysteines are also the most and least abundant amino acids (Tyagi et al., 2020). The frequency of the codon AUU encoding isoleucine is the highest, whereas that of the codon CGC encoding arginine is the lowest (Table S3). Codon usage bias is also measured by calculating the relative synonymous codon usage (RSCU), which represents the ratio between the usage frequency of a specific codon and the expected frequency. If the RSCU value is >1, the use frequency of code is higher than the expected frequency, and RSCU value of <1 indicates the opposite result (Sharp and Li, 1987). Thirty preferred (RSCU > 1) synonymous codons are detected, indicating that these codons are preferentially used in coding amino acids. Additionally, we found that only the codons encoding Trp (UGG) and Met (AUG) amino acids have no bias (RSCU = 1); however, other codons have an obvious bias in *B. balsamifera* (Figure S2). Intriguingly, except UUG, all preferentially used codons end with A/U. This result agrees with that observed in other species (Munyao et al., 2020; Tyagi *et al.*, 2020), which shows that the dominant codon contains more A or U in codon selection and the high proportion of A/U is the driving force of deviation.

### Repeat sequence analysis

Simple sequence repeats (SSRs), known as microsatellite DNA, are molecular markers often used in phylogenetics, identification, and population genetic studies of plant species because they are highly reliable, reproductive, and polymorphic (Kaur et al., 2015). This study identified 53 SSRs in the cp genome of *B. balsamifera* (mononucleotide, dinucleotide, trinucleotide, and tetranucleotide; Table S4). Mononucleotide repeats (79.37%) are the most abundant, whereas the trinucleotide repeats (3.17%) are the least (Table S4), indicating that mononucleotide repeats contribute more to genetic variations than other SSRs. Furthermore, we found that the content of A or T in 4 SSRs was the highest, which illustrates that SSRs usually comprise poly-A and poly-T, rarely tandem guanine (G) and cytosine (C), thereby contributing to the AT richness of the cp genome. Additionally, 40 long repeats are identified in the *B. balsamifera* genome (Table S5), including 18 forward repeats (F), 21 palindromic repeats (P), and one reverse repeat (R). The long repeat sequences range from 30 to 59 bp, and most of them are concentrated in the range of 30-49 bp. They mainly exist in the intergenic spacers; however, *psaB*, *psbA*, *ycf3*, *ndhB*, *ycf2*, *and ycf1* genes comprise the majority that contains long repeats. The long repeats are also detected more in LSC than in SSC and IR regions. Similar results are also noted in other *Asteraceae*, indicating that long repeats exist in noncoding regions (Tyagi et al., 2020). Overall, repeat sequences can reshape the cp genome and reveal the genetic diversity among different species.

### Comparative genome analysis

**Figure 2 -:**
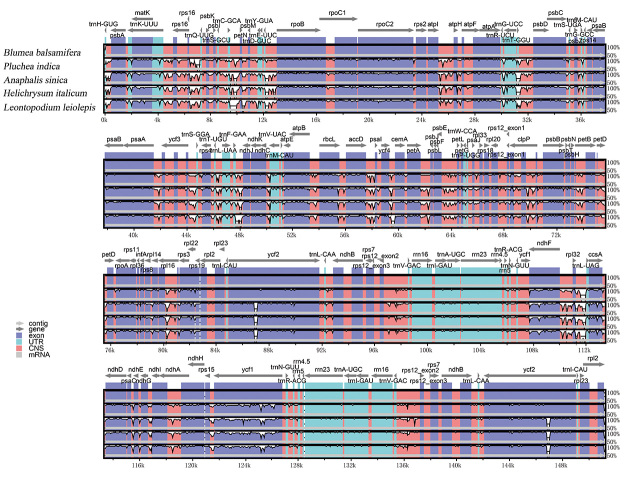
The cp genomes of *Blumea balsamifera* and four other *Asteraceae* plants were compared using the mVista program with *Blumea balsamifera* as the reference. The arrow indicates the position and orientation of the gene. The Y-axis represents the percentage of identity among genome sequences (50%-100%).

Comparative analysis of DNA sequences helps in identifying important gene regions and functional genes. The mVISTA is a common tool for comparative genome analysis and helps in quickly identifying the conserved regions of DNA sequences (Ratnere and Dubchak, 2009). By comparing the whole cp genome of *B. balsamifera* and other four *Asteraceae* plants (*A. sinica*, *L. leiolepis*, *H. italicum*, and *P. indica*), we found that the conservation of the IR region among the five species was higher than that of the LSC and SSC regions. Genetic variability was mainly concentrated in the intergenic or noncoding region (Figure 2). The coding regions with a large variation in the five chloroplast genomes are *matK*, *atpB*, *accD*, *cemA*, *rpoA*, *ycf2*, *ndhF*, *ccsA*, *ndhI*, *ndhH*, *rps15*, and *ycf1* (Figure 2). The regions with large differences are widely used to study population and plant system genetics (Kim and Jansen, 1995). For example, *matK* has been widely used in the core universal DNA barcode of species, whereas *ycf1* is used in plant phylogeny and DNA barcode research (Dong et al., 2015; Yang et al., 2017). Additionally, *accD*, *rpoA*, *ccsA*, and *ndhF* have significant differences, which can be used to study plant development systems (Li et al., 2019). We can study these regions further to develop more DNA barcode markers for the identification of *Blumea* species.

### Phylogenetic analysis

**Figure 3 -:**
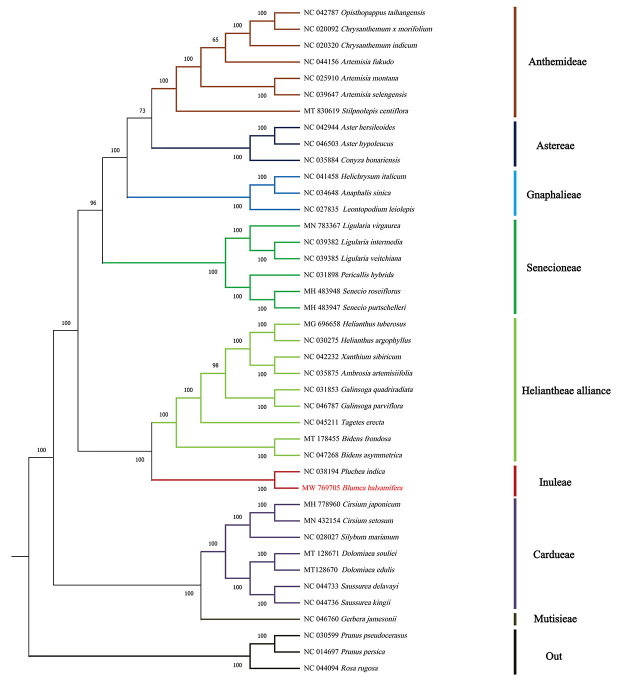
The maximum likelihood (ML) phylogenetic tree based on the complete chloroplast genome sequence was constructed with three species of *Rosaceae* as outgroup. The number above the tree node indicates the bootstrap support value.

With the rapid development of sequence technology, several cp genomes of plants have been revealed, making it possible to explore the relationship between plant phylogeny and evolution from the molecular perspective (Tonti-Filippini et al., 2017). For the determination of the phylogenetic status and evolutionary relationship of *B. balsamiferae* in *Asteraceae*, the complete cp genome sequences of 37 reported *Asteraceae* species were selected to construct the maximum likelihood (ML) phylogenetic tree and three species of the *Rosaceae* family were considered as the outgroup (Figure 3). Phylogenetic analysis shows that *Asteraceae* species form a monophyletic group and are divided into several subgroups (Anthemideae, Astereae, Gnaphalieae, Senecioneae, Heliantheae alliance, Inuleae, Cardueae, and Mutisieae). *B. balsamifera* and *P. indica* are clustered into the same branch with a bootstrap value of 100%, and both belonged to Inuleae. The genetic relationship between Inuleae and Heliantheae alliance is close, whereas that between Inuleae and Astereae is far, which is consistent with the results of Tyagi et al. (2020). Our classification supports Anderberg’s 1991 and 2005 classification results from a genomic perspective. This study fills the gap in the research of the cp genome of *Blumea* plants, which provides abundant information regarding the taxonomic study of this genus in *Asteraceae* plants.

## Conclusions

Cp genomes have been widely considered an informative and valuable resource for molecular marker development and phylogenetic reconstruction in plant species. In this study, the complete cp genome of *B. balsamifera* was reported for the first time. The cp genome of *B. balsamifera* is 151,170 bp in size, and its genome structure, gene number, and gene sequence are similar to those of other *Asteraceae* plants. We found that there are more A or U in the preferred codon. Combining the results with previous studies, we speculate that the high content of A or U is an important reason for the deviation of coding genes. Furthermore, 53 SSRs were found, which can be used for studies on population genetics and genetic breeding of *Blumea*. Comparison of the whole cp genome of *B. balsamifera* with that of other *Asteraceae* species indicates that the IR region is more conservative than the SSC and LSC regions. Finally, a phylogenetic tree was constructed based on the complete chloroplast genomes of 37 *Asteraceae* species and three *Rosaceae* species, confirming that *B. balsamifera* had the closest relationship with *P. indica* from the molecular viewpoint and revealed the position of *B. balsamifera* in *Asteraceae*. Thus, the complete cp genome of *B. balsamifera* provides valuable genetic information for this genus and lays a foundation for identifying and studying population evolution in *Asteraceae* species.

## Supplementary material

The online material available for this article contains the following:

Table S1 - List of 40 chloroplast genomes used in the phylogenetic analysis

Table S2 - Features of the chloroplast genomes of *Blumea balsamifera*

Table S3 - Codon usage in the *Blumea balsamifera* chloroplast genomes

Table S4 - Types and amount of SSRs of *Blumea balsamifera*

Table S5 - Analysis of long sequence repeats in the *Blumea balsamifera* chloroplast genome

Figure S1 - Ratio of amino acids and stop codons in the cp genome of *Blumea balsamifera*

Figure S2 - RSCU histogram of *Blumea balsamifera.* The upper figure shows the sum of RSCU values of codons in amino acids. The following blocks represent all codons that encode each amino acid.
