# Supplementary material for: Chloroplast genome characteristics and phylogenetic analysis of the medicinal plant Blumea balsamifera (L.) DC
Source: Genet Mol Biol. 2021 Nov 15;44(4):e20210095. doi: 10.1590/1678-4685-GMB-2021-0095 (PMC8628730; doi:10.1590/1678-4685-GMB-2021-0095)
Supplement: Table S1 - [file 1415-4757-GMB-44-4-e20210095-s1.pdf]

Genetics and Molecular Biology
DOI: https://doi.org/10.1590/1678-4685-GMB-2021-0095

Supplementary Material to "Chloroplast Genome Characteristics and Phylogenetic Analysis of the Medicinal Plant *Blumea balsamifera* (L.) DC"

Table S1 - List of 40 chloroplast genomes used of in the phylogenetic analysis.

Serial number	Species name	Taxon	Family	Accesion number
1	*Artemisia fukudo*	Anthemideae	Asteraceae	NC_044156.1
2	*Artemisia montana*			NC_025910.1
3	*Artemisia selengensis*			NC_039647.1
4	*Chrysanthemum indicum*			NC_020320.1
5	*Chrysanthemum morifolium*			NC_020092.1
6	*Opisthopappus taihangensis*			NC_042787.1
7	*Stilpnolepis centiflora*			MT830619.1
8	*Conyza bonariensis*	Astereae		NC_035884.1
9	*Aster hypoleucus*			NC_046503.1
10	*Aster hersileoides*			NC_042944.1
11	*Anaphalis sinica*	Gnaphalieae		NC_034648.1
12	*Helichrysum italicum*			NC_041458.1
13	*Leontopodium leiolepis*			NC_027835.1
14	*Tagetes erecta*	Heliantheae alliance		NC_045211.1
15	*Ambrosia artemisiifolia*			NC_035875.1
16	*Bidens frondosa*			MT178455.1
17	*Bidens asymmetrica*			NC_047268.1
18	*Galinsoga parviflora*			NC_046787.1
19	*Galinsoga quadriradiata*			NC_031853.1
20	*Helianthus tuberosus*			MG696658.1
21	*Helianthus argophyllus*			NC_030275.1
22	*Xanthium sibiricum*			NC_042232.1
23	*Pluchea indica*	Inuleae		NC_038194.1
24	*Ligularia veitchiana*	Senecioneae		NC_039385.1
25	*Ligularia intermedia*			NC_039382.1
26	*Ligularia virgaurea*			MN783367.1
27	*Pericallis hybrida*			NC_031898.1
28	*Senecio roseiflorus*			MH483948.1
29	*Senecio purtschelleri*			MH483947.1
30	*Cirsium setosum*	Cardueae		MN432154.1

Serial number	Species name	Taxon	Family	Accesion number
31	*Cirsium japonicum*			MH778960.1
32	*Dolomiaea souliei*			MT128671.1
33	*Dolomiaea edulis*			MT128670.1
34	*Saussurea kingii*			NC_044736.1
35	*Saussurea delavayi*			NC_044733.1
36	*Silybum marianum*			NC_028027.1
37	*Gerbera jamesonii*	Mutisieae		NC_046760.1
38	*Prunus pseudocerasus*	Out		NC_030599.1
39	*Prunus persica*			NC_014697.1
40	*Rosa rugosa*			NC_044094.1
