# Supplementary material for: Chloroplast genome characteristics and phylogenetic analysis of the medicinal plant Blumea balsamifera (L.) DC
Source: Genet Mol Biol. 2021 Nov 15;44(4):e20210095. doi: 10.1590/1678-4685-GMB-2021-0095 (PMC8628730; doi:10.1590/1678-4685-GMB-2021-0095)
Supplement: Table S2 - [file 1415-4757-GMB-44-4-e20210095-s2.pdf]

Genetics and Molecular Biology
DOI: https://doi.org/10.1590/1678-4685-GMB-2021-0095

Supplementary Material to “Chloroplast Genome Characteristics and Phylogenetic Analysis of the Medicinal Plant *Blumea balsamifera* (L.) DC”

Table S2 - Features of the chloroplast genomes of *Blumea balsamifera*.

Category	Attributes	Descriptions
Construction of cp genome	Total cp DNA size (bp)	151,170
	LSC size (bp)	82,740
	SSC size (bp)	18,466
	IR size (bp)	49,964
Gene number	Number of total genes	130
	Number of Protein-coding genes	85
	Number of rRNA genes	8
	Number of tRNA genes	37
	Number of duplicate genes	17
GC content	GC content of genome (%)	37.50%
	GC content of LSC (%)	35.80%
	GC content of SSC (%)	31.10%
	GC content of IR (%)	43.00%
