# Supplementary material for: Chloroplast genome characteristics and phylogenetic analysis of the medicinal plant Blumea balsamifera (L.) DC
Source: Genet Mol Biol. 2021 Nov 15;44(4):e20210095. doi: 10.1590/1678-4685-GMB-2021-0095 (PMC8628730; doi:10.1590/1678-4685-GMB-2021-0095)
Supplement: Table S3 - [file 1415-4757-GMB-44-4-e20210095-s3.pdf]

Genetics and Molecular Biology
DOI: https://doi.org/10.1590/1678-4685-GMB-2021-0095

Supplementary Material to "Chloroplast Genome Characteristics and Phylogenetic Analysis of the Medicinal Plant *Blumea balsamifera* (L.) DC"

Table S3 - Codon usage in the *Blumea balsamifera* chloroplast genomes.

Codon	Amino acid	Count	RSCU	Codon	Amino acid	Count	RSCU
UUU	F (Phe)	976	1.31	UCU	S (Ser)	592	1.77
UUC	F (Phe)	517	0.69	UCC	S (Ser)	301	0.9
UUA	L (Leu)	853	1.84	UCA	S (Ser)	418	1.25
UUG	L (Leu)	587	1.27	UCG	S (Ser)	169	0.51
CUU	L (Leu)	612	1.32	CCU	P (Pro)	419	1.52
CUC	L (Leu)	181	0.39	CCC	P (Pro)	198	0.72
CUA	L (Leu)	389	0.84	CCA	P (Pro)	323	1.17
CUG	L (Leu)	160	0.35	CCG	P (Pro)	160	0.58
AUU	I (Ile)	1085	1.47	ACU	T (Thr)	534	1.64
AUC	I (Ile)	426	0.58	ACC	T (Thr)	237	0.73
AUA	I (Ile)	699	0.95	ACA	T (Thr)	410	1.26
AUG	M (Met)	626	1	ACG	T (Thr)	124	0.38
GUU	V (Val)	512	1.47	GCU	A (Ala)	626	1.77
GUC	V (Val)	158	0.45	GCC	A (Ala)	230	0.65
GUA	V (Val)	529	1.52	GCA	A (Ala)	418	1.18
GUG	V (Val)	196	0.56	GCG	A (Ala)	143	0.4
UAU	Y (Tyr)	799	1.62	UGU	C (Cys)	208	1.43
UAC	Y (Tyr)	189	0.38	UGC	C (Cys)	82	0.57
UAA	*	48	1.69	UGA	*	15	0.53
UAG	*	22	0.78	UGG	W (Trp)	466	1
CAU	H (His)	463	1.52	CGU	R (Arg)	353	1.34
CAC	H (His)	146	0.48	CGC	R (Arg)	98	0.37
CAA	Q (Gln)	712	1.52	CGA	R (Arg)	350	1.32
CAG	Q (Gln)	225	0.48	CGG	R (Arg)	120	0.45
AAU	N (Asn)	996	1.56	AGU	S (Ser)	406	1.22
AAC	N (Asn)	282	0.44	AGC	S (Ser)	116	0.35
AAA	K (Lys)	1030	1.47	AGA	R (Arg)	488	1.85
AAG	K (Lys)	374	0.53	AGG	R (Arg)	176	0.67
GAU	D (Asp)	853	1.62	GGU	G (Gly)	571	1.3
GAC	D (Asp)	203	0.38	GGC	G (Gly)	192	0.44
GAA	E (Glu)	994	1.48	GGA	G (Gly)	687	1.56
GAG	E (Glu)	347	0.52	GGG	G (Gly)	307	0.7

*stop codon; RSCU = 1 (not biased); RSCU > 1 (high biased); RSCU < 1 (less biased)
