# Supplementary material for: Chloroplast genome characteristics and phylogenetic analysis of the medicinal plant Blumea balsamifera (L.) DC
Source: Genet Mol Biol. 2021 Nov 15;44(4):e20210095. doi: 10.1590/1678-4685-GMB-2021-0095 (PMC8628730; doi:10.1590/1678-4685-GMB-2021-0095)
Supplement: Table S4 - [file 1415-4757-GMB-44-4-e20210095-s4.pdf]

Genetics and Molecular Biology
DOI: https://doi.org/10.1590/1678-4685-GMB-2021-0095

Supplementary Material to "Chloroplast Genome Characteristics and Phylogenetic Analysis of the Medicinal Plant *Blumea balsamifera* (L.) DC"

Table S4 - Types and amount of SSRs of *Blumea balsamifera*

SSR type	Repeat unit	Amount	Ratio (%)
Mono	A/T	49	77.78
	C/G	1	1.59
Di	AT/AT	4	6.35
Tri	AAG/CTT	2	3.17
Tetra	AAAG/CTTT	1	1.59
	AAAT/ATTT	5	7.94
	AATC/ATTG	1	1.59
