# Supplementary material for: Chloroplast genome characteristics and phylogenetic analysis of the medicinal plant Blumea balsamifera (L.) DC
Source: Genet Mol Biol. 2021 Nov 15;44(4):e20210095. doi: 10.1590/1678-4685-GMB-2021-0095 (PMC8628730; doi:10.1590/1678-4685-GMB-2021-0095)
Supplement: Table S5 - [file 1415-4757-GMB-44-4-e20210095-s5.pdf]

Genetics and Molecular Biology
DOI: https://doi.org/10.1590/1678-4685-GMB-2021-0095

Supplementary Material to "Chloroplast Genome Characteristics and Phylogenetic Analysis of the Medicinal Plant *Blumea balsamifera* (L.) DC"

Table S5 - Analysis of long sequence repeats in the *Blumea balsamifera* chloroplast genome

ID	Repeat start 1	Type	Size (bp)	Repeat start 2	Locus	Region
1	377	P	30	413	IGS	LSC
2	4434	R	30	4443	IGS	LSC
3	8488	F	32	35100	IGS	LSC
4	8490	P	30	44790	IGS; *trnS-GGA*	LSC
5	10653	P	50	10653	IGS	LSC
6	11912	F	32	11975	IGS	LSC
7	35102	P	30	44790	IGS; *trnS-GGA*	LSC
8	38286	F	32	40510	*psaB*; *psaA*	LSC
9	43295	P	41	137172	*ycf3*; IGS	LSC, IRa
10	43295	F	41	96697	*ycf3*; IGS	LSC, IRb
11	43297	P	39	115746	*ycf3*; *ndhE*	LSC, SSC
12	43300	P	35	140221	*ycf3*; *ndhB*	LSC, IRa
13	43300	F	35	93654	*ycf3*; *ndhB*	LSC, IRb
14	46349	P	52	46349	IGS	LSC
15	66052	P	30	135994	IGS	LSC, IRa
16	66052	F	30	97886	IGS	LSC, IRb
17	72791	P	48	72791	IGS	LSC
18	89925	P	30	143919	*ycf2*	IRb, IRa
19	89925	F	30	89961	*ycf2*	IRb
20	89928	P	45	143919	*ycf2*	IRb, IRa
21	89928	F	45	89946	*ycf2*	IRb
22	89946	P	45	143937	*ycf2*	IRb, IRa
23	89961	P	30	143955	*ycf2*	IRb, IRa
24	93654	P	35	115747	*ndhB*; *ndhE*	IRb, SSC
25	96699	P	39	115746	IGS; *ndhE*	IRb, SSC
26	97577	P	40	136272	IGS	IRb, IRa
27	97577	F	40	97598	IGS	IRb
28	97598	P	40	136293	IGS	IRb, IRa
29	105633	P	30	128215	IGS	IRb, IRa
30	105633	F	30	105665	IGS	IRb
31	105665	P	30	128247	IGS	IRb, IRa
32	108409	F	36	108460	*ndhF*	SSC

ID	Repeat start 1	Type	Size (bp)	Repeat start 2	Locus	Region
33	115746	F	39	137172	*ndhE*; IGS	SSC, IRa
34	115747	F	35	140221	*ndhE*; *ndhB*	SSC, IRa
35	123666	P	47	123666	*ycf1*	SSC
36	128215	F	30	128247	IGS	IRa
37	136272	F	40	136293	IGS	IRa
38	143919	F	30	143955	*ycf2*	IRa
39	143919	F	45	143937	*ycf2*	IRa
40	143933	F	31	143951	*ycf2*	IRa

F forward; P palindrome; R reverse; IGS intergenic spacer region
