# Supplementary material for: Chloroplast genome characteristics and phylogenetic analysis of the medicinal plant Blumea balsamifera (L.) DC
Source: Genet Mol Biol. 2021 Nov 15;44(4):e20210095. doi: 10.1590/1678-4685-GMB-2021-0095 (PMC8628730; doi:10.1590/1678-4685-GMB-2021-0095)

Genetics and Molecular Biology
DOI: https://doi.org/10.1590/1678-4685-GMB-2021-0095

Supplementary Material to "Chloroplast Genome Characteristics and Phylogenetic Analysis of the Medicinal Plant *Blumea balsamifera* (L.) DC"

Figure S2 - RSCU histogram of *Blumea balsamifera.* The upper figure shows the sum of RSCU values of codons in amino acids. The following blocks represent all codons that encode each amino acid.

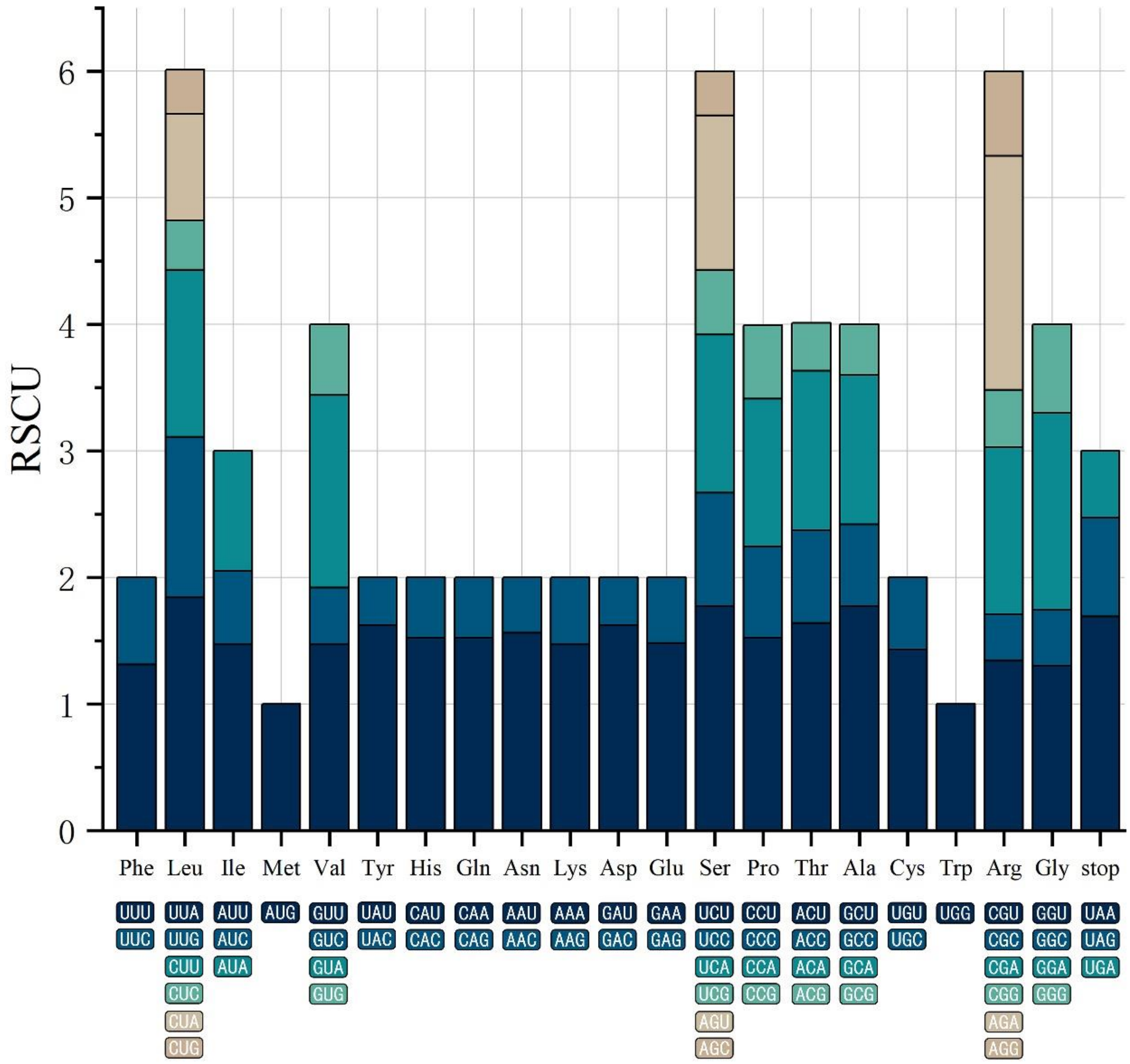

Supplement: Figure S2 - [file 1415-4757-GMB-44-4-e20210095-s7.pdf]
